# Experimental Study on a “Snake-Type” Vibration Cutting Method for Cutting Force and Cutting Heat Reductions

**DOI:** 10.3390/biomimetics4030057

**Published:** 2019-08-13

**Authors:** Xiangyu Zhang, Zhenlong Peng, Deyuan Zhang

**Affiliations:** 1 Beijing Advanced Innovation Center for Biomedical Engineering, Beihang University, Beijing 100091, China; 2 School of Mechanical Engineering and Automation, Beihang University, Beijing 100091, China; 3 Institute of Bionic and Micro-Nano Systems, Beihang University, Beijing 100091, China

**Keywords:** cutting, force, heat, surface integrity, snake, Odontomachus monticola

## Abstract

Cutting is the foundation of manufacturing in industry. The main cutting objects include metals, ceramics, glasses, compositions, and even biological materials such as tissues and bones. The special properties of each material such as hardness, ductility, brittleness, and heat conductivity lead to either a large cutting force or a high cutting temperature. Both of these factors result in poor machinability due to rapid tool wear or break or unsatisfactory surface integrity of the material finishing surface using the conventional cutting (CC, conventional cutting) types. In nature, snakes have their own way of reducing heat accumulation on their body when moving on the hot desert surface. They move forward along an “S”-type path, so that the bottom of their body separates from the desert intermittently. In this way, the separation interval both reduces the cutting heat accumulations and effectively achieves cooling by allowing the air to go through. In addition, the acceleration of *Odontomachus monticola*’s two mandibles when striking a target can reach 71,730 g m/s^2^ within 180 ms, which can easily break the target surface by the transient huge impact. Therefore, based on a snake’s motion on the desert surface and *Odontomachus monticola*’s striking on the target surface, respectively, an ultrasonic-frequency intermittent cutting method, also called “snake-type” vibration cutting (SVC, snake-type vibration cutting), was proposed in this study. First, its bionic kinematics were analyzed, then the SVC system’s design was introduced. Finally, cutting experiments were conducted on a common and typical difficult-to-cut material, namely titanium alloys. Cutting force, cutting temperature, and the surface integrity of the material finishing surface were measured, respectively. The results demonstrated that, compared to conventional cutting methods, SVC achieved a maximum of 50% and 30% reductions of cutting force and cutting temperature, respectively. Moreover, the surface integrity was improved both in surface roughness and residual stress state.

## 1. Introduction

Cutting is the foundation of manufacturing in industry. It includes the fields of food, energy, navigation, aviation, aeronautics, and biomedical science and touches our daily lives. Using a designed material removal trajectory, a desired structure with satisfactory surface quality can be fabricated by cutting. Generally, the main cutting objects include metals, ceramics, glasses, compositions, and even biological materials such as tissues and bones. However, the special properties of each material such as hardness, ductility, brittleness, and heat conductivity lead to either a large cutting force or a high cutting temperature. As the crown of manufacturing, aviation engines comprise typical difficult-to-cut components. They consist mostly of titanium alloys, super alloys, and composites [1] that have high strength, high hardness, and low heat conductivity. Therefore, during the cutting process, this leads to rapid tool wear [2], extremely low cutting tool life [3], and chip burning [4]. All of these mentioned disadvantages result in a poor surface quality and low cutting efficiency.

Many attempts have been made in research studies to reduce the cutting force and cutting heat. Some typical methods are cutting parameter [5,6], cutting tool material [7,8], coating material [9,10], cutting tool geometry optimizations [11], cooling [12,13,14], and intermittent cutting [15]. With respect to optimizations, even though surface quality can be improved by reducing the cutting force and cutting heat, the application range of these methods is limited because only certain specific parameter combinations can achieve the cutting force and cutting heat reductions. Meanwhile, numerous experiments must be conducted to find the appropriate ones. As for cooling, when high-pressure, oil-mist, cryogenic, and minimum quantity lubrication (MQL) are applied, the cutting force and heat reductions can reach a maximum value of 38% and 62%, respectively [16]. As for surface integrity, microhardness can be 10% and 18.3% lower for ferrite and pearlite when turning with MQL, respectively, whereas the crumple zone can also be half depth as that without cooling [17]. Moreover, if oil-based cooling and lubricating liquids are applied, this can effectively reduce friction and surface roughness [18]. However, during the cutting process, the force and heat sources cannot be cooled or lubricated owing to the continuous contact between the tool rake face, chip, and workpiece surface. In this regard, there is still some room for improvement if the contact zone is opened. Intermittent cutting was then proposed to solve this problem. As a typical intermittent cutting, vibration cutting was firstly proposed in the 1950s. When a small vibration was added to the tip of the cutting tool insert, researchers found huge benefits beyond their imagination, that is, a cutting force reduction of 75% when turning steels [19] and of 98% when cutting aluminum [20]. In addition, surface roughness was reduced by 60% and ferrous metal cutting was achieved using diamond tools [21]. However, these benefits were obtained under conditions of an extremely lower cutting speed, also sacrificing cutting efficiency. A critical cutting speed exists which is related to the vibration amplitude and frequency [22]. When the cutting speed increases and approaches the critical value, the separation effect between the tool and workpiece becomes weaker and finally vanishes. Thus, all benefits become weaker and finally vanish correspondingly. Therefore, in order to achieve cutting force and cutting heat reductions and use the tool impact during the material removal process, highly efficient intermittent cutting methods must be developed.

**Figure 1:**
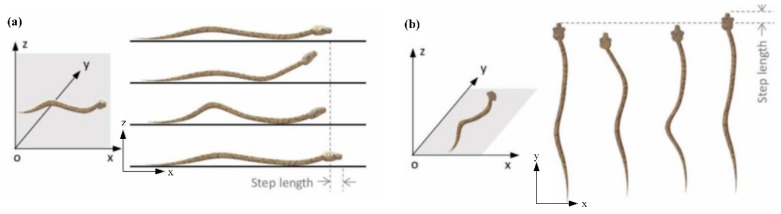
Mechanisms of a snake’s locomotion. (**a**) Snake’s concertina locomotion and (**b**) snake’s serpentine locomotion [25] (with permission).

In nature, snakes have attracted researchers’ attention owing to their locomotion behaviors [23,24]. Shown in Figure 1 [25], snakes have concertina and serpentine locomotion at the same time. In Figure 1a, very few points on the snake’s abdomen are in contact with the support surface. In addition, when snakes move forwards, the contact points are changing all the time. Similarly, in Figure 1b, snakes move as a curve rather than a straight line. In this way, certain kinds of snakes can endure high surface temperatures and live in deserts [26]. The periodical separation of their body from the surface and the changing contact points can effectively make their body cool down, whereas the transient impact with a specific angle from the moving direction on the surface can act as a tight support and its driving force. Therefore, the locomotion of a snake can guide us in finding an innovative separation style. Unlike the vibration direction which is parallel to the cutting direction, the snake-type vibration is vertical to its cutting direction, either in the radial (top-to-bottom) direction (Figure 1a) or in the axial (side-to-side) direction (Figure 1b).

**Figure 2:**
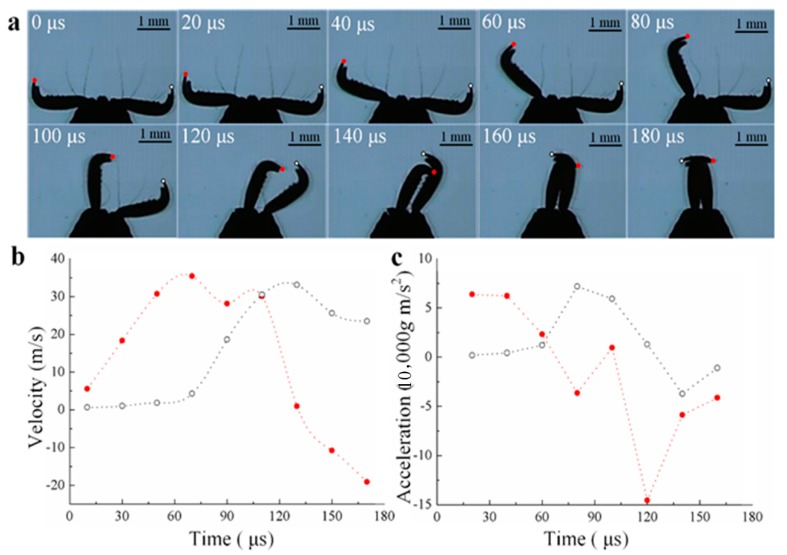
Kinematics of the *Odontomachus monticola* strikes. (**a**) High-speed video images of a strike process (red-filled circle and white open circle represent the right and left mandibles, respectively), 50,000 fps. (**b**) The velocities of both mandibles, which can reach 35.42 m/s on the right mandible (red-filled circle). (**c**) The accelerations of both mandibles, which can reach 71,730 g m/s^2^ on the left mandible (white open circle) [30].

As for the transient impact, mantis shrimp [27] and *Odontomachus bauri* [28,29] have already been observed to reach a maximum speed of 23 m/s and 35–64 m/s, accelerating about 10,400× *g* when striking the targets, respectively. However, the radius of the mantis shrimp’s striking hammer is about 2.5 mm, which is much larger than the cutting contact zone. Therefore, we chose a variety of trap-jaw ants living in China, *Odontomachus monticola*, for our study due to the small radius of its striking tip (about 25 μm). The kinematics analysis of the striking process is given in Figure 2 [30]. In Figure 2a, we see that the striking time is no longer than 50 μs and that the maximum velocity and acceleration can reach 35.42 m/s and 71,730 g m/s^2^, as is shown in Figure 2b,c, respectively. In this regard, only ultrasonic-frequency vibration may have the potential to achieve such a huge impact within a tiny interval.

As mentioned above, inspired by a snake’s locomotion and the impact parameters of *Odontomachus monticola,* a “snake-type” vibration cutting (SVC, snake-type vibration cutting) method was developed for this study. The design and principle of the SVC method are given and calculated at the beginning of this paper. Then, the experiments set up to verify the feasibility of the separation effect in the SVC process using a transient cutting force signal [31] are presented. Based on the separation effect, the cutting force and cutting temperature reductions are validated by measuring using dynamometers [32] and tool–workpiece thermocouples [33]. Finally, surface integrity is discussed to prove the feasibility of the SVC method in cutting difficult-to-machine materials.

## 2. Materials and Methods

### 2.1. Design and Principle of the SVC Method

**Figure 3:**
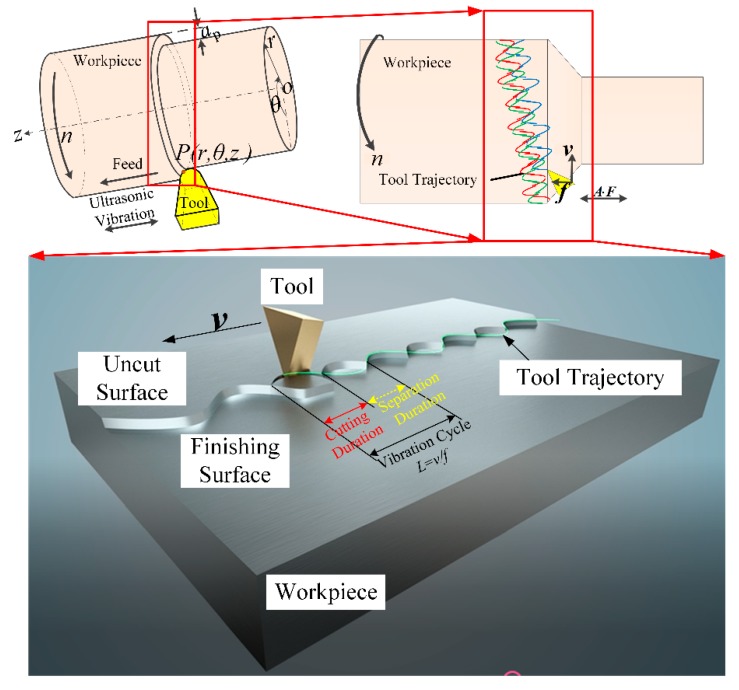
Schematics of the “snake-type” vibration cutting (SVC, snake-type vibration cutting) method.

Based on the typical machining process, namely turning, SVC is presented in Figure 3. A periodical vibration parallel to the axial direction of the workpiece or spindle is added onto the tooltip. In this way, the tool moves like the snake’s serpentine locomotion on the workpiece surface, while on the normal direction of the uncut surface, the tool also moves like the snake’s concertina locomotion. During one vibration cycle, the duration when the tool gets engaged into the workpiece is defined as the cutting duration, and the duration when the tool gets disengaged from the workpiece is defined as the separation duration. In the cutting duration, the tool shears the material and generates the chip, whereas in the separation duration, the chip is broken at the moment when the tooltip leaves the workpiece surface and a transient tiny interval between the cutting tool and workpiece forms.

In the axial direction, also known as the *z* direction, the tool motion trajectory is given by Z=Asin(2πFt), where *Z*, *A*, and *F* are the tooltip displacement in the *z* direction, the tool vibration amplitude, and the vibration frequency, respectively. In this way, it is easy to calculate the impact velocity *V_I_* and impact acceleration *a_I_* as $V_{I}=\dot{Z}=2\pi FA\cos\left(2\pi Ft\right)$ and $a_{I}=\ddot{Z}=-4\pi^{2}F^{2}A\sin\left(2\pi Ft\right)$.

As mentioned above, when the impact duration is set at about 50 μs, the corresponding vibration frequency is about 20,000 Hz. Therefore, piezoelectric ceramics are used to stimulate the ultrasonic vibration. Generally, the vibration amplitude after the magnification of the horn embedded in the tool shank reaches about 20 μm. Based on these data, the impact acceleration reaches about 32,000 g m/s^2^, which is half of the maximum value produced by *Odontomachus monticola*.

This differs from the existing critical cutting speed in the previous intermittent cutting process that confines the separation effect within an extremely low cutting speed range [22]. It is easily seen in Figure 3 that the separation effect using the SVC method has nothing to do with the cutting speed. If the separation effect must occur, we need only guarantee an appropriate phase between two adjacent cutting tool trajectories while the feed rate is no greater than the vibration amplitude. The phase is related to the vibration frequency and workpiece rotary speed, *n* (r/min). Therefore, the SVC method can achieve high-speed intermittent machining, and a ratio of the cutting moment in one vibration cycle is used to describe this separation degree, namely, *t_c_/T*.

### 2.2. Experimental Design and Setup

**Figure 4:**
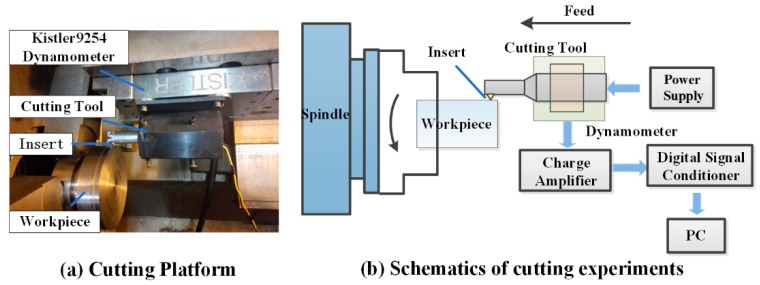
Experiment platform.

The experiment platform was set up as is shown in Figure 4. All experiments were designed and conducted on a CNC lathe (HASS SL40, Oxnard, CA, USA). Cemented carbide triangular cutting tools were chosen (Zhuzhou. Co., Zhuzhou, Hunan, China, TCMT110204) with a tool nose radius of 0.4 mm, rake angle of 0 degrees, flank angle of 7 degrees, and TiAlN coating. The cutting tool was fixed onto a dynamometer (Kistler 9254A, Winterthur, Switzerland) to obtain the average cutting force signal and moved along the axial direction. The vibration was stimulated by the power supply generated by the laboratory itself. The real resonant vibration frequency was 21,588 Hz, whereas the non-loaded vibration amplitude was 20 μm.

Titanium-4Aluminum-6Vanadium (Ti-4Al-6V) alloys were chosen as the workpiece due to their extremely low heat conductivity and the severe adhesive effect on the cutting tool, both of which generally resulted in rapid tool failure and poor surface integrity in the conventional cutting (CC) method. The diameter of the workpiece was 120 mm.

First, the separation effect of SVC was verified through the transient cutting force signal obtained by a high-frequency responding force sensor (PCB208C02) [31]. Then, average cutting force signals were obtained by the dynamometer which was linked to a charge amplifier (Kistler 5070, Winterthur, Switzerland) and a digital signal conditioner (Kistler 5509A, Winterthur, Switzerland). At the same time, the cutting temperature was measured using a tool–workpiece thermocouple [33]. In the measuring process, the cutting zone was regarded as the hot end of the thermocouple and a calibration curve between the thermovoltage and temperature was observed. Finally, surface integrity was measured. Surface roughness was measured using a roughometer (MarSurf PS1, Esslingen, Germany), surface topography was observed using a three-dimensional white light interference profiler (ZYGONewView, Connecticut, USA), and surface residual stress was detected using a prism electronic speckle pattern interferometry drilling measurement apparatus (Stresstech Group, Helsinki, Finland).

For the transient cutting force measurement, the cutting parameters were set as follows: cutting speed, 200 m/min; feed rate, 0.005 mm/r; and depth of cut, 0.05 mm. The phase was adjusted by altering the spindle speed slightly so as to change the ratio of vibration frequency and spindle rotary speed.

As for the average cutting force measurement, the cutting parameters are listed in Table 1. Cutting speed was set at a start value of 200 m/min, which was far beyond the cutting speed of the CC method. All the feed rate values were less than the vibration amplitude in order to achieve the separation effect. In addition, the depth of cut was set in the precision machining range. The cutting ratio was set as 0.55 through a real-time feedback control system [34].

With respect to the cutting temperature measurement, a cutting speed of 400 m/min was added to describe the changing trend of the temperature reduction. When measuring the surface roughness, Ra and Rz, feed rate was chosen as the only variable and ranged from 0.005 mm/r to 0.02 mm/r. In this way, the cutting state was transited from intermittent cutting to continuous cutting (the feed rate value is equal to the vibration amplitude). Moreover, with respect to surface topography, a low-speed large feed rate combination (50 m/min and 0.02 mm/r) was added to clearly observe the snake motion trajectories. Finally, the finishing surface under a parameter combination (200 m/min, 0.1 mm, and 0.005 mm/r) was chosen for the surface residual stress measurement because of the optimal cutting force and cutting temperature reductions.

Regular cooling fluid (embedded in the lathe system) was used throughout all the experiments in order to obtain satisfactory cooling or lubrication conditions.

## 3. Results and Discussion

### 3.1. Cutting Force

**Figure 5:**
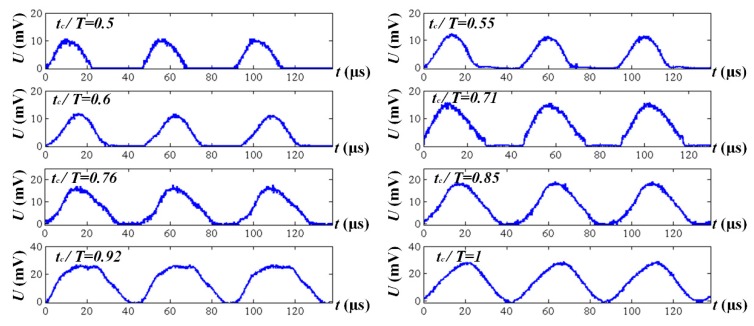
Transient cutting force signals of different cutting ratios.

**Figure 6:**
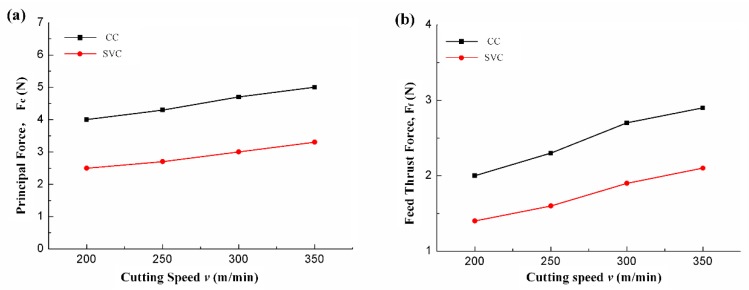
Average (**a**) principal and (**b**) feed thrust forces vs. cutting speed (feed rate, 0.005 mm/r; depth of cut, 0.025 mm).

**Figure 7:**
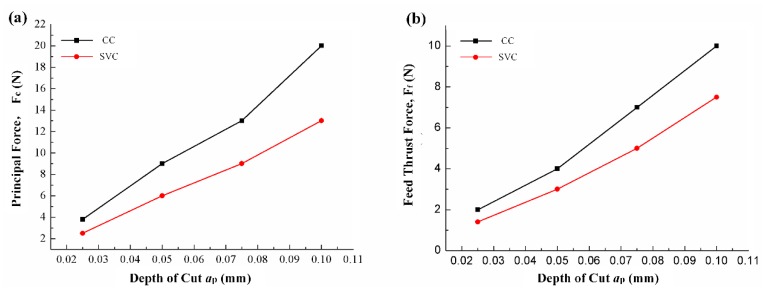
Average (**a**) principal and (**b**) feed thrust forces vs. depth of cut (feed rate, 0.005 mm/r; cutting speed, 200 m/min).

**Figure 8:**
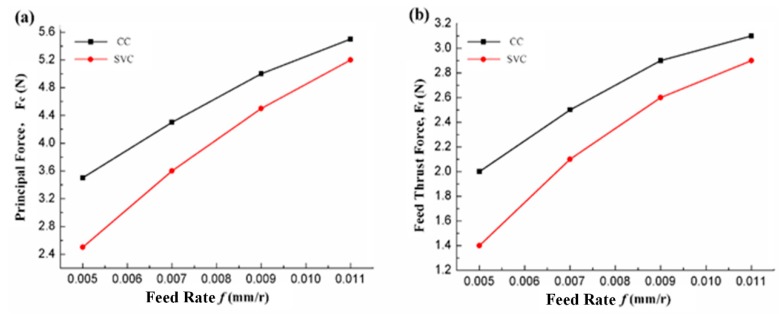
Average (**a**) principal and (**b**) feed thrust forces vs. feed rate (depth of cut, 0.025 mm; cutting speed, 200 m/min).

Transient cutting force signals of different cutting ratios are presented in Figure 5. When the tool separates from the workpiece, the cutting force signals drop to the zero line. Thus, the average cutting force of SVC is reduced due to the existence of the separation effect. The experiment results are clearly shown in Figure 6, Figure 7 and Figure 8.

As the cutting speed increases, both the average cutting forces of CC and SVC are increased simultaneously. However, a maximum cutting force reduction of 38% and 55% for principal and feed thrust force is achieved, respectively. Taking the cutting ratio of 0.5 in Figure 5, for example, the transient impact force approaches approximately 10 N. However, the separation interval and the impact force signal shape both finally result in a small average cutting that is approximately 2.8 N (see Figure 7b). In this regard, we can assume that, during the SVC process, an extremely transient impact occurs which happens more quickly than the cutting duration. Similar to *Odontomachus monticola*’s strike process, the transient impact during each vibration cycle leads to a much better material breaking than the regular cutting force of CC. After the extra impact, it becomes easy to tear up and remove the material. Therefore, unlike traditional material removal, the material is removed intermittently during the SVC process by continuous ultrasonic-frequency impulse.

As the depth of cut increases, the cutting force increases significantly, even though the separation effect has nothing to do with the depth of cut once the feed rate value is determined. However, as the depth of cut increases, the energy for material removal is increased accordingly. Therefore, the cutting force reduction ratio decreases.

As is known from the separation conditions and as is shown in Figure 5, once the feed rate value increases, the cutting ratio increases accordingly. In this regard, the peak of the cutting force signal increases significantly. Moreover, when the shortening of the separation duration is taken into account simultaneously, the average cutting force of SVC is greatly enhanced and the cutting force reduction ratio decreases.

### 3.2. Cutting Temperature

**Figure 9:**
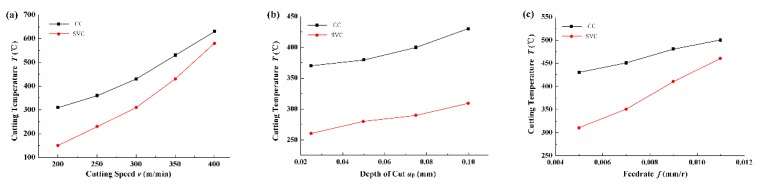
Cutting temperature vs. (**a**) cutting speed (feed rate, 0.005 mm/r; depth of cut, 0.1 mm), (**b**) depth of cut (cutting speed, 300 m/min; feed rate, 0.005 mm/r), and (**c**) feed rate (cutting speed, 300 m/min; depth of cut, 0.1 mm).

As demonstrated in Figure 9, cutting temperature is reduced to a maximum value of 50% when the cutting speed, feed rate, and depth of cut are 200 m/min, 0.005 mm/r, and 0.1 mm, respectively.

In Figure 9a, the cutting temperature increase is approximately exponential as the cutting speed increases. When the cutting speed is 400 m/min, the reduction ratio is close to 8.3%. Figure 9b illustrates the relationship between the depth of cut and cutting temperature. As is clearly seen, the reduction ratio keeps stable and the change in depth of cut has no obvious influence. However, as the feed rate increases, the separation effect also weakens, the cutting temperature of SVC increases rapidly, and the reduction ratio decreases.

### 3.3. Surface Integrity

**Figure 10:**
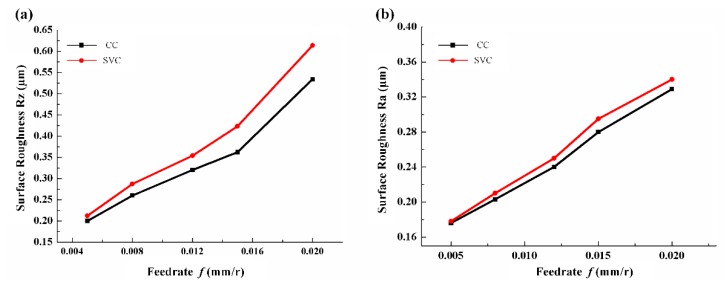
Surface roughness (**a**) R_z_ and (**b**) R_a_ vs. feed rate (cutting speed, 200 m/min; depth of cut, 0.05 mm).

As is shown in Figure 10, the surface roughness of SVC and CC keeps the same level for any given feed rate condition. On the one hand, due to the cutting force reduction in SVC, the system stiffness and allowable depth of cut are enhanced [35]. For that point, the surface quality of SVC is better than that of CC. However, on the other hand, in CC, the tool just moves along the cutting direction and keeps engaged in the workpiece, and the cutting thickness remains the same all the time. On the contrary, due to the vibration tool trajectory, the surface of SVC will form peaks when the tool gets engaged in the workpiece and dimples when it reaches the maximum cutting thickness. Taking these two factors together, the measured results show in Figure 10a that the highest peak and dimple value (R_z_) of SVC is higher than that of CC, whereas in Figure 10b, the average values (R_a_) remain almost the same. However, if the feed rate value is restricted within an appropriate range in which there is a relatively large separation effect, the peaks and dimples are removed periodically by the adjacent cutting tool trajectories. Thus, the surface roughness of SVC can be controlled within the precision machining range, namely, R_a_ < 0.4 μm.

**Figure 11:**
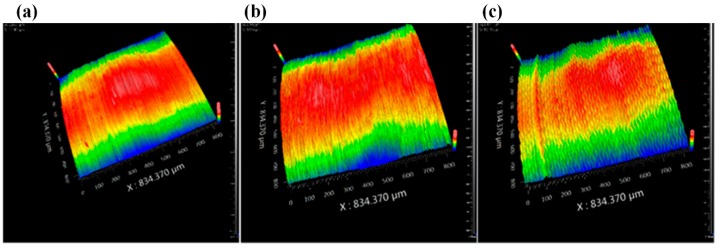
Surface topography (depth of cut, 0.05 mm) (**a**) CC, *f* = 0.005 mm/r, *V* = 200 m/min, (**b**) SVC, *f* = 0.005 mm/r, *V* = 200 m/min and (**c**) SVC, *f* = 0.02 mm/r, *V* = 5 0 m/min.

The surface topographies are shown in Figure 11. It can be seen that, in CC, the surface consists of regular groove arrays. They are formed by the tooltip shape and feed rate. The 3D parameters of Figure 11a are Sa = 1.555 μm and Sq = 1.845 μm. As a comparison, the surface in SVC under the same cutting conditions consists of periodical curves. The intersections of the adjacent cutting tool trajectories result in a special cutting unit once the separation condition is satisfied. However, during the high-speed machining process, the shape of one vibration cycle is long and thin. The length in the cutting direction is about 167 μm and the width in the feed direction is about 20 μm in the situation presented in Figure 11b. Altogether with the cutting trajectory intersection, the snake-type motion is difficult to distinguish. Therefore, a low cutting speed and large feed rate combination is observed in Figure 11c. When the feed rate reaches 0.02 mm/r, the separation effect no longer occurs. In addition, the low cutting speed makes the cutting length shorter than in the high-speed situation. Thus, a clear snake-type locomotion is observed on the finishing workpiece surface. The 3D parameters of SVC in these two situations are Sa = 1.386 μm, 1.628 μm, Sq = 1.642 μm, 1.934 μm, respectively. It can be clearly seen that the surface roughness of both SVC and CC are at the same level.

**Figure 12:**
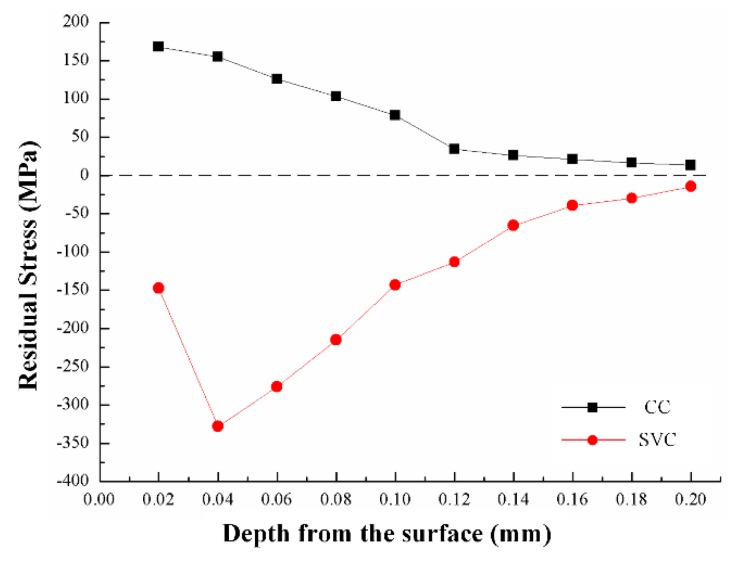
Surface residual stress (cutting speed, 200 m/min; depth of cut, 0.1 mm; and feed rate, 0.005 mm/r).

Based on the cutting force impact and cutting temperature reductions, the surface residual stress state is transferred from the tensile state to a compressive one. This transfer indicates a potential material fatigue improvement. As is clearly shown in Figure 12, due to the higher cutting temperature in CC than SVC, the residual stress of CC in the workpiece surface is tensile and becomes smaller in deeper positions, whereas the residual stress of SVC is compressive and reaches a maximum value of 347 MPa (minus sign means compressive state) caused by the cutting tool impact just as the tool cuts into the workpiece. The position is 0.04 mm depth from the surface. Then, as the depth increases, the influence of both cutting temperature and tool impact decreases and the residual stress of both methods decreases and tends back to balanced states. The influence depth is about 50% deeper than the depth of cut. If the cut goes deeper into the sample, the residual stresses of both methods tend to go to the opposite state and fluctuate to release the stress within.

**Figure 13:**
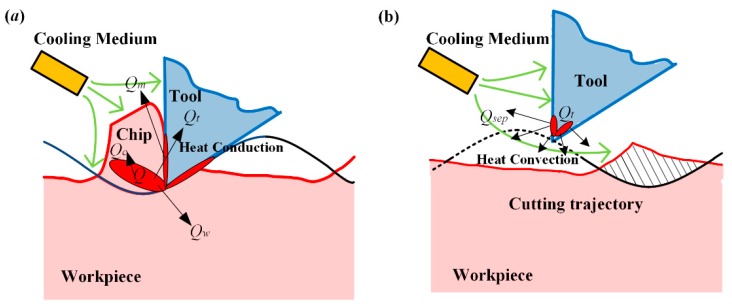
Schematics of the cutting mechanism in (**a**) cutting duration and (**b**) separation duration.

The reasons for such benefits derived from SVC can be ascribed to the separation effect. On the one hand, the separation effect leads to a cutting temperature decrease (Figure 9), and on the other hand, it causes a flank face extrusion on the finishing workpiece surface. The schematics of the cutting mechanism are illustrated in Figure 13. In the cutting duration shown in Figure 13a, once the cutting tool gets engaged into the workpiece, the cutting force is generated by the shearing of the workpiece and the friction in the interfaces between the cutting tool and chip. Once the cutting force is generated, cutting heat generates simultaneously. In this process, the material is torn up and the cutting temperature rises owing to the cutting heat accumulation. The cutting zone at that moment is closed where the cutting tool and workpiece are tightly in contact. Therefore, the cutting medium cannot reach the force and heat sources to ensure lubrication and cooling. However, when the cutting tool gets disengaged from the workpiece in the separation duration shown in Figure 13b, the small transient gap between the cutting tool and the workpiece lets the cooling medium into the cutting zone. In this way, a heat convection between the heat source and cooling medium takes place. Therefore, the cutting temperature is reduced compared to CC because of no heat generation on the one hand and strong heat convection on the other hand. When the next cutting duration arrives, the cutting zone is lubricated to a certain level due to the remaining cooling medium on the workpiece surface.

As for the surface residual stress, the tool motion trajectory leads to a flank face impact on the finishing workpiece surface during the cutting process. In this way, the mechanical influence is enhanced due to the extrusion effect. At the same time, the cutting temperature reduction decreases the thermal deformation of the surface workpiece. Therefore, the surface residual stress state can be transferred from tensile state to compressive state.

## 4. Conclusions

A “snake-type” vibration cutting method was proposed in this paper. Because of an extra ultrasonic vibration on the tooltip, the traditional continuous machining process was turned into an intermittent machining process. In the cutting duration, a transient cutting impact leads to broken chips, whereas in the separation duration, the gap between the cutting tool and the workpiece provides a path for the cooling medium penetration. Compared to CC and traditional vibration cutting methods, this innovative periodical machining process achieves certain advantages as follows:

(1) A high cutting speed (200–400 m/min) can be achieved compared to traditional vibration cutting methods (<60 m/min).
(2) A maximum cutting force and cutting temperature reduction of about 50% can be achieved compared to CC when the cutting speed is 200 m/min.
(3) SVC can achieve a compressive surface residual stress (347 MPa) and same surface roughness level as that of CC (R_a_ < 0.4 μm).

However, even though SVC can achieve such benefits, this research is still in a preliminary step. First, compared to traditional vibration cutting methods, there is still enough room for improvement regarding the surface quality of SVC. In this preliminary step, the surface quality only reaches the same level as that achieved using CC. Second, the mechanism of cutting force and heat temperature reduction has not been discovered and the discussions are based on kinematics and process experiments. Therefore, the improvement demonstrated in this paper is thought to be conservative. Moreover, the influence of changing vibration amplitude and waveform must be carefully investigated because vibration parameters can directly influence cutting states. Finally, although SVC provides an effective high-speed machining method for difficult-to-cut materials, it is worthwhile to expect further research studies about the comprehensive understanding of these cutting force and cutting temperature reductions and the highly effective use of the separation effect to broaden the application range (milling, drilling, grinding, etc.).

## Figures and Tables

**Table 1:** Cutting parameters of average cutting force measurement.

Parameters	Values
Cutting speed, m/min	200, 250, 300, 350
Feed rate, mm/r	0.005, 0.007, 0.009, 0.011
Depth of cut, mm	0.025, 0.05, 0.075, 0.1
Cutting fluid	Regular cooling fluid
